# Polymethylmethacrylate Pulmonary Embolism Following Vertebroplasty

**DOI:** 10.7759/cureus.17314

**Published:** 2021-08-19

**Authors:** Scott D Myers, Mitchell Streiff, Adam R Dulberger, Max American, Christopher D Sanders

**Affiliations:** 1 Department of Diagnostic Radiology, David Grant Medical Center, Fairfield, USA; 2 Department of Diagnostic Radiology, Ponce Health Sciences University, Ponce, USA; 3 Department of Interventional Radiology, David Grant Medical Center, Fairfield, USA

**Keywords:** polymethylmethacrylate embolism, percutaneous vertebroplasty, pulmonary embolism, computed tomography angiography, interventional radiology, conservative management

## Abstract

Polymethylmethacrylate (PMMA) is a commonly used substrate in vertebroplasty procedures. Well-known for its dependable strength and relative lack of toxic side effects, PMMA administration is useful for the stabilization of vertebral bodies in the setting of common spinal pathologies such as osteoporosis. Unfortunately, as the popularity of vertebroplasty has increased, so has the incidence of a potentially lethal complication of the procedure, PMMA pulmonary embolism. Extravasation of PMMA from the vertebral body into the adjacent vasculature can provide a route through which PMMA may travel until it becomes lodged in the pulmonary vasculature, thereby forming a PMMA pulmonary embolism. While the vast majority of PMMA embolism cases are relatively mild, others are severe and demand swift recognition and potentially life-saving intervention. Despite the increasing incidence of PMMA embolism, a clear algorithm for management does not yet exist. Controversy abounds regarding the most effective strategies to diagnose and manage patients with PMMA embolism. Described is a case of delayed diagnosis of a PMMA embolism in a patient who underwent percutaneous vertebroplasty for an osteoporotic vertebral body fracture. Multiple visits to the emergency department (ED) for chest discomfort or cough after the vertebroplasty eventually led to cross-sectional imaging that revealed the diagnosis. Her acute symptoms resolved with conservative management. Given that her final outcome was positive with no long-term morbidity, the aim of this report is to explore the current treatment algorithms for PMMA embolism and to consider whether or not this patient would have been managed differently had the correct diagnosis been uncovered earlier.

## Introduction

Polymethylmethacrylate (PMMA) pulmonary embolism is a potential complication of vertebroplasty that can either be asymptomatic or manifest with nonspecific pulmonary symptoms. PMMA, an artificial cement, is the agent of choice in most vertebroplasty procedures [1]. Unfortunately, even when administered with a sound technique, PMMA can undergo extravasation and become lodged in the pulmonary vasculature causing pulmonary embolism [2]. The severity of PMMA emboli can range from completely asymptomatic to life-threatening. Although the vast majority of patients with PMMA emboli are asymptomatic or develop only mild pulmonary symptoms, some cases are acutely life-threatening. Considering the grave potential of PMMA emboli, providers should keep this diagnosis high on the list of differential diagnoses when a patient presents with respiratory symptoms in the context of recent vertebroplasty [3]. Management of PMMA emboli is highly controversial and there is not a well-established management algorithm. Options for management range from symptomatic care only to heparin therapy to thrombectomy in the most severe of cases [2]. Early suspicion and recognition of symptomology due to PMMA embolism are key for a timely diagnosis. Radiologic imaging can play a paramount role in determining the specific localization and extent of PMMA emboli, knowledge which can ultimately guide effective management. Described is a case of PMMA pulmonary embolism in a patient to which the diagnosis was not initially attributed. The purpose of this case report is to detail the possible presentation of PMMA pulmonary embolism and discuss the complication’s etiology, sequelae, and imaging characteristics in an effort to assist healthcare professionals in managing this niche patient population appropriately.

## Case presentation

**Figure 1:**
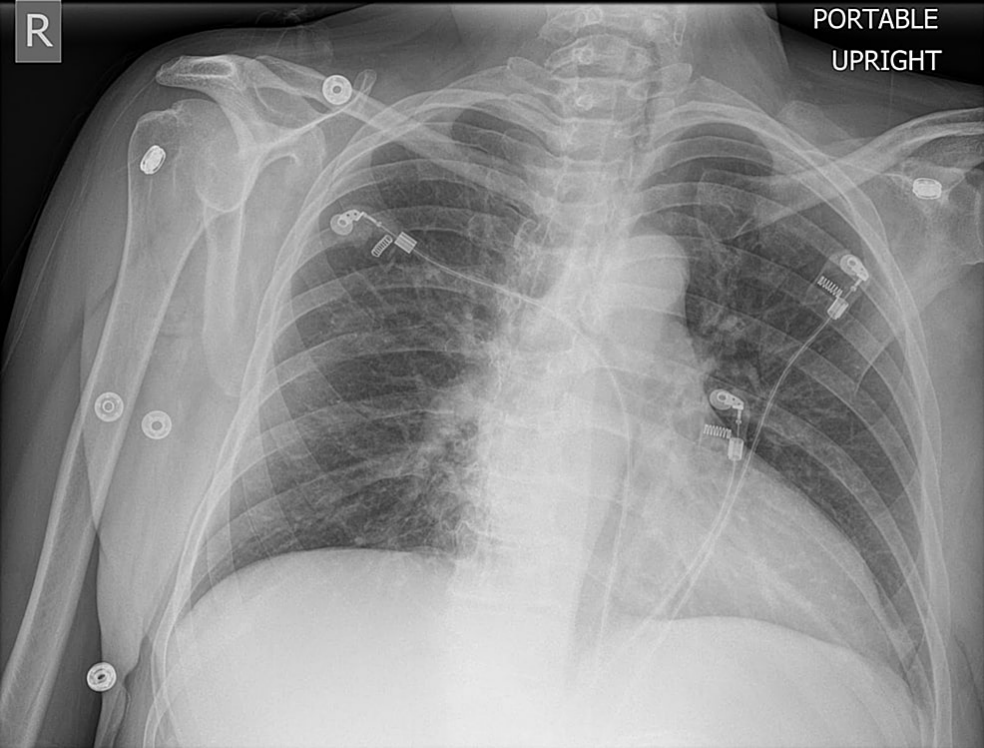
CXR negative for any acute cardiopulmonary process. CXR: chest x-ray.

The patient is a 66-year-old female with multiple medical problems, most notably alcohol dependence. She presents to the emergency department (ED) frequently for falls or altered mental status related to intoxication. Earlier in the year, she was brought to an ED by ambulance after being found on the ground. Physical examination revealed tenderness to palpation over the lower thoracic and upper lumbar spine. The patient was subsequently found to have a lumbar spine compression fracture, likely related to her trauma in the setting of osteopenia, however, chest x-ray (CXR) was negative for any acute cardiopulmonary process (Figure 1). 

She was worked up in a timely manner as an outpatient for management of a symptomatic compression fracture of L1 and shortly thereafter underwent a percutaneous vertebroplasty. 

**Figure 2:**
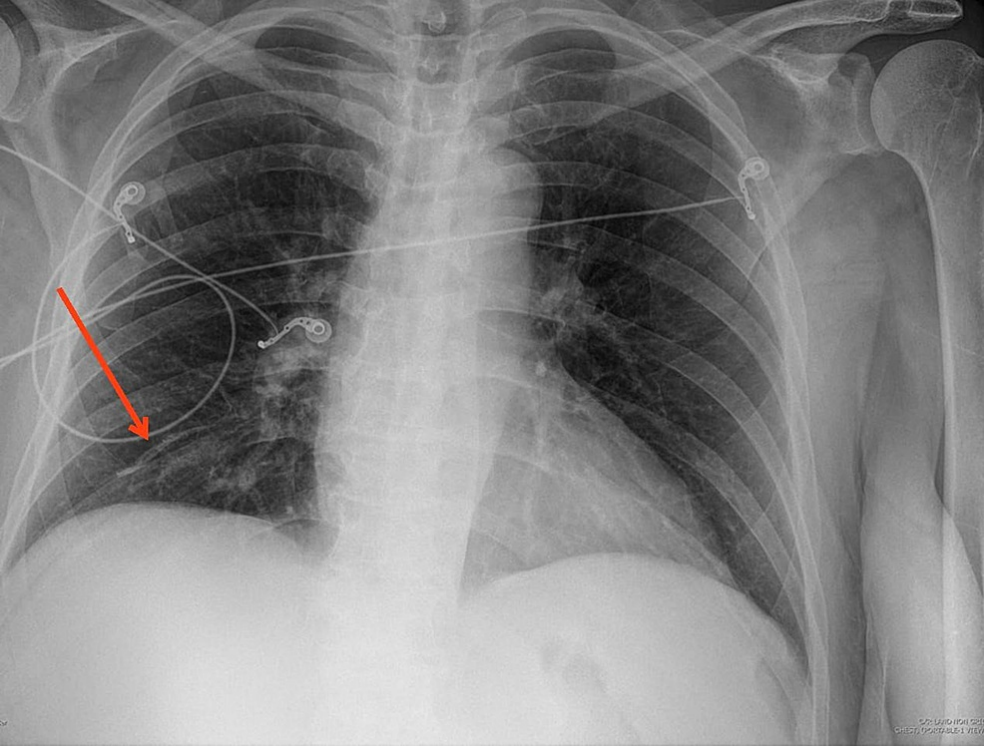
New-onset linear density of the right lower lobe following vertebroplasty, thought to be subsegmental atelectasis.

While she initially experienced improvement of her back pain, several weeks after her procedure, she presented to the ED for mild chest pain. A CXR was ordered and interpreted as showing a new linear density of the right lower lobe, presumed to be subsegmental atelectasis (Figure 2); however, an abnormality to explain the patient's symptoms was not identified, and she was consequently discharged.

Within a few weeks, she returned again to the ED with similar complaints of chest discomfort and dry cough. A repeat CXR was again interpreted as linear atelectasis of the right lower lobe, unchanged from the findings in the previous CXR.

**Figure 3:**
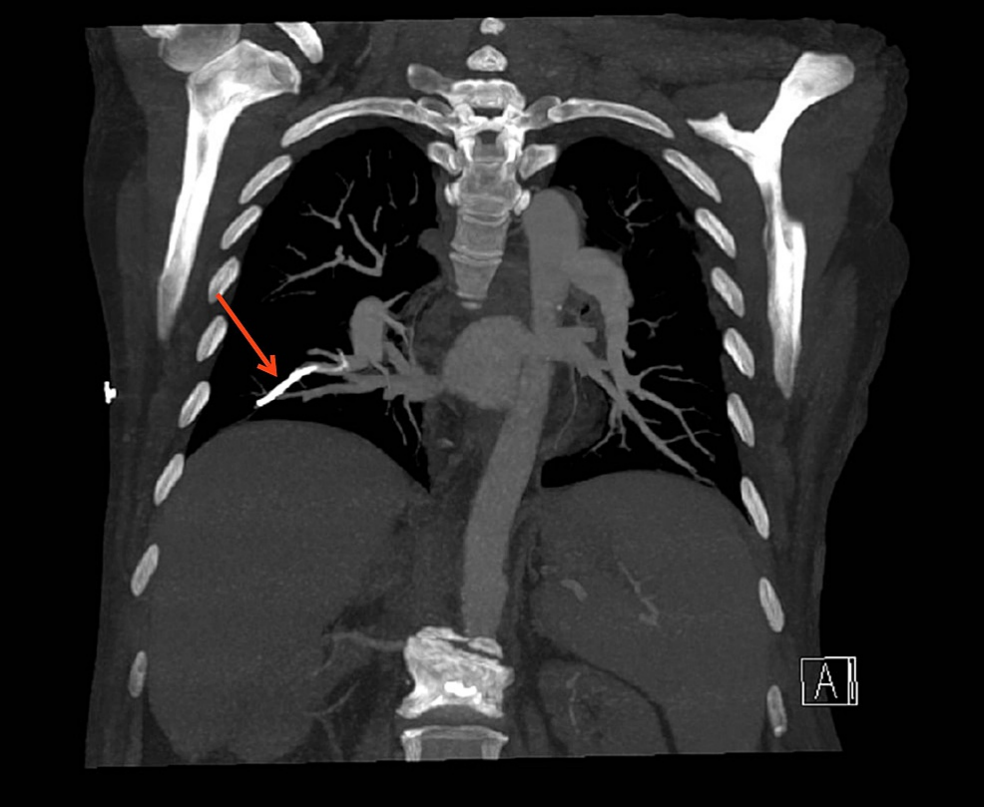
CTA of chest showing high density material in the vertebral column and subsegmental pulmonary artery of the right lower lobe. CTA: computed tomography angiography

The patient then underwent a computed tomography angiography (CTA) of the chest. The CTA was negative for any acute pulmonary parenchymal process; however, it offered novel insight into the conclusions drawn from the previous CXRs. What had previously been deemed lower lobe atelectasis was better delineated as high density material within a subsegmental right lower lobe pulmonary artery (Figure 3). 

No intrinsic thrombus or embolism was documented surrounding the high density material or elsewhere in the pulmonary arteries. The CTA report noted this high density material, however, it was erroneously described as retained contrast in a pulmonary vein. This observation represented a type 2 error-identification, but incorrect diagnosis. The patient was discharged again. 

Almost a month after her CTA, the patient returned to the hospital again, this time for a mild cough. The radiologist reading the CXR noted the right lower lobe linear density and determined it was too dense to represent simple atelectasis. With the patient’s history of vertebroplasty in mind, a retrospective evaluation of the CTA suggested an alternate diagnosis, that of a cement embolism. The average Hounsfield unit (HU) of the linear hyperdensity was >1200, consistent with a PMMA embolism, the cement used for vertebroplasty. Considering that the PMMA embolism had been present for several months and the cross-sectional imaging did not show an intrinsic thrombosis or evidence of parenchymal infarction, no further recommendation for anticoagulation was made. 

## Discussion

For nearly three decades, percutaneous vertebroplasty has been utilized to help with symptomatic vertebral height loss related to various etiologies including hemangiomas, osteoporotic deformities, and metastatic fractures [4,5]. Percutaneous vertebroplasty is a relatively safe and minimally invasive procedure that has a high success rate of mitigating back pain related to microfractures, compression fractures, or other osseous structural abnormalities of a vertebral body [6]. Under fluoroscopic guidance, the vertebral body marrow space is accessed via a transpedicular approach with a bone needle before instilling PMMA. The use of real-time imaging aids in both placement of the bone needle(s) as well as ensuring the PMMA distributes appropriately without extravasation. There have been multiple reviews regarding access via a single pedicle versus both pedicles and which approach is safer or has fewer complications, but no true consensus has been made [7].

PMMA is an artificial cement that when mixed properly achieves a toothpaste-like consistency so that it can be appropriately introduced into the vertebral marrow space. It is widely used in vertebroplasty procedures due to its inert nature, dependable strength, and lack of toxicity [1]. Proper preparation of the material is of paramount importance; too thin and the likelihood of extravasation increases, too thick and the administration is more difficult. Once in place, this artificial cement stabilizes the vertebral body, often leading to pain reduction. Ideally, the cement should be distributed, be structurally supportive in the anterior two-thirds of the vertebral body, while avoiding excessive filling [6]. Higher volumes of the cement are associated with increased post-procedural vertebral body strength, however, higher volumes also increase the probability of cement extravasation. Therefore, the fundamental challenge of vertebroplasty is the administration of the correct amount of cement in the correct anatomical location, so as to confer the bone-strengthening properties without promoting any cement extravasation sequelae [2]. 

As with most procedures, complications include infection, bleeding, and damage to adjacent structures. Specific to vertebroplasty, feared complications involve damage to nerve roots or the spinal cord, new or worsening fracture(s), advancement of the bone needle beyond the anterior vertebral cortex, and PMMA embolization secondary to cement extravasation. Cement extravasation is the most common complication of vertebroplasty; however, significant adverse events occur in very few (1%) cases in which there is extravasation [3]. Extravasation can lead to spinal cord and nerve injury, intervertebral disc injury, and cardiac or pulmonary embolization. 

**Figure 4:**
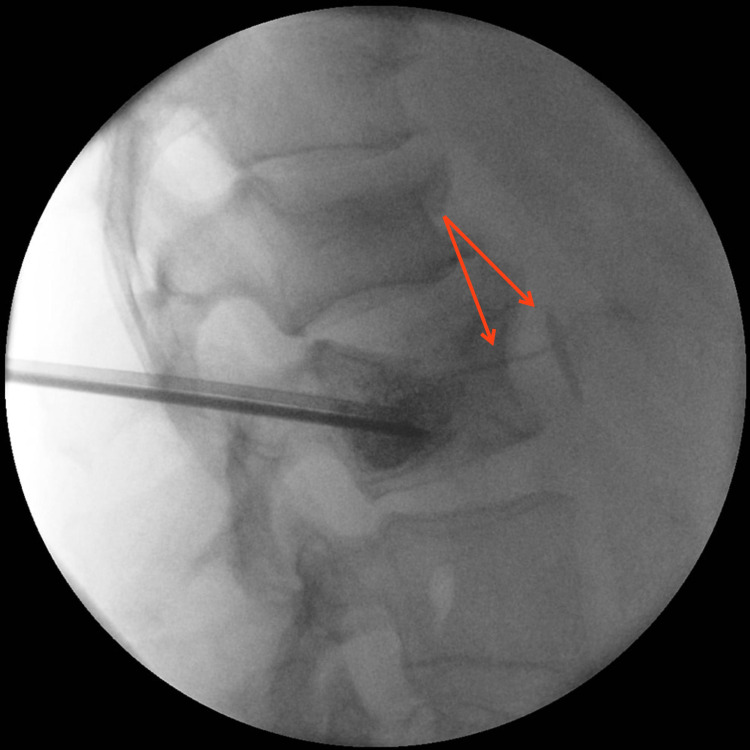
Fluoroscopy image demonstrating opacification of a paravertebral vein during the vertebral PMMA injection. PMMA: polymethylmethacrylate.

In this case, the patient initially presented a few weeks after her vertebroplasty and then again, several months later, with various nonspecific symptoms to include cough and atypical chest pain. Given the suspected pulmonary etiology of these symptoms, providers ordered imaging of the chest that was ultimately determined to show a PMMA embolism in an anterior-basal subsegmental artery of the right lower lobe. Retrospective analysis of the fluoroscopy images taken during the vertebroplasty demonstrates opacification of a paravertebral artery during the vertebral PMMA injection (Figure 4), consistent with the initial seeding of a pulmonary embolism. 

While the presence of a PMMA extravasation is clear in the fluoroscopy imaging, it is questionable whether or not the patient’s symptoms were attributable to the embolism that eventually formed, given that the symptoms were self-limited, delayed in presentation, and never focally associated with the right lower chest. This case demonstrates that good outcomes can be obtained with conservative therapies, without the risks of anticoagulation use or invasive treatments. Despite the delay in diagnosis, this patient ultimately received an appropriate treatment course.

Incidental segmental and subsegmental pulmonary artery PMMA emboli are not uncommon. Additionally, PMMA emboli found distal to the segmental artery level are almost always clinically insignificant. The review of the literature revealed a broad incidence of PMMA emboli ranging between 2% and 26% based on the imaging modality used [3]. The true incidence of pulmonary emboli formation secondary to PMMA extravasation has proven exceptionally difficult to quantify, given that the vast majority of patients are asymptomatic and consequently do not undergo any type of post-procedural imaging that would reveal emboli presence [3,8]. That said, several factors have been shown to be related to an increase in the incidence of clinically significant pulmonary emboli development. Delivery of PMMA volumes greater than 9 mL and performance of vertebroplasty in the setting of a compression fracture secondary to a tumor, rather than secondary to osteoporosis, have both been shown to be associated with an increase in the incidence of PMMA emboli [8,9]. 

As more and more vertebroplasties are performed in the management of osteoporosis, radiologists should be aware of the imaging findings associated with PMMA emboli. Due to its relatively high density, when compared to the normal lung parenchyma, PMMA can be visible on CXR. High-density opacities branching throughout an area of pulmonary arterial distribution can be suggestive of PMMA embolism and should be recorded in the radiology report [10]. While CXR can be used as a screening tool for PMMA embolism, chest CT is a much more sensitive modality for the diagnosis. Findings on CT are comparable to those potentially visible on CXR, branching high-density opacities in the distribution of pulmonary arterial vasculature, however, CT generally offers clearer visualization of these findings [11].

Treatment for PMMA emboli is highly controversial. Some have suggested that therapy should be based on clinical symptoms and the severity of such symptoms at presentation. Other authors have proposed management based on the size and location of the PMMA embolism. Given that the vast majority of these emboli are small, peripheral, and asymptomatic, most patients are treated with close observation and follow-up, rather than any specific medical management. Patients with small, incidentally identified PMMA emboli who present with mild chest discomfort or cough have commonly been treated with conservative management, however, more recent recommendations encourage a course of heparin for three to six months. Some authors have recommended that PMMA emboli that are centrally located, large, or found in patients who present severe symptoms of respiratory failure, should likely warrant more aggressive treatment, even thrombectomy in the gravest of cases [2,12,13]. Given the lack of evidence-based data, it is still challenging to support the early use of anticoagulation in cases of an inert PMMA embolism. This patient offers potential insight into the reliability of conservative management, rather than anticoagulation, specifically in the case of a small and peripheral PMMA embolism. She ended up recovering over time without further complications, suggesting that it is not unreasonable to consider conservative management in patients who are mildly symptomatic as long as there is no evidence of marked respiratory distress or pulmonary infarction. Ultimately, not enough data has been obtained to declare definitive management. 

## Conclusions

It is important to know that PMMA embolization during percutaneous vertebroplasty is a relatively common complication. As more and more vertebroplasties are performed in the United States, the incidence of PMMA embolization will invariably continue to rise. Recognizing the imaging findings associated with PMMA emboli can help predict the clinical significance, if any. Not only is the identification of a pulmonary PMMA embolism important, but proper interpretation, for which the radiologist can play a role, is paramount in guiding appropriate management. Given that the current literature has demonstrated that the vast majority of PMMA emboli are small, peripheral, and asymptomatic, conservative therapy is all that will be required in most cases. That said, for cases in which patients exhibit more severe symptoms, anticoagulation or thrombectomy are not unreasonable treatment options. This case demonstrated that even in a mildly symptomatic patient, conservative management did not lead to intrinsic thrombus formation, respiratory distress, or worsening clinical presentation. Determination of a more evidence-based treatment algorithm will evolve over time, but for now most therapeutic decisions will be made on a case-by-case basis.
